# Adsorption of N, He and Ne on CGe nanoribbons for sensing and optoelectronic applications

**DOI:** 10.1098/rsos.231836

**Published:** 2024-03-27

**Authors:** Hoang Van Ngoc

**Affiliations:** ^1^ Center for Forecasting Study, Institute of Southeast Vietnamese Studies, Thu Dau Mot University, Thu Dau Mot, Binh Duong province, Vietnam

**Keywords:** CGe nanoribbons, nitrogen, helium, neon, adsorption configurations

## Abstract

Research into nanomaterials yields numerous exceptional applications in contemporary science and technology. The subject of this investigation is a one-dimensional nanostructure, six atoms wide, featuring hydrogen-functionalized edges. The theoretical foundation of this study relies on Density Functional Theory (DFT) and is executed through the utilization of the Vienna Ab initio Simulation Package (VASP). The outcomes demonstrate the stability of adsorption configurations, along with the preservation of a hexagonal honeycomb lattice. The pristine configuration, characterized by a wide bandgap, is well-suited for optoelectronic applications, whereas adsorption configurations find their application in gas sensing. Nitrogen (N) adsorption transforms the semiconducting system into a semimetallic one, with the spin-up state showing semiconductor characteristics and the spin-down state exhibiting metallic attributes. The intricate multi-orbital hybridization is explored through the analysis of partial states. While the pristine system remains non-magnetic, N adsorption introduces a magnetic moment of 0.588 μ_B_. The examination of charge density differences indicates a significant charge transfer from N to the CGe substrate surface. Optical properties are systematically investigated, encompassing the dielectric function, absorption coefficient and electron–hole density. Notably, the real part of the dielectric function exhibits negative values, a result that holds promise for future communication applications.

## 1. Introduction

Through the relentless dedication of scientists, new materials have been unearthed during the eras of industrialization, catering to the pragmatic needs of humanity. Modern devices are becoming progressively more sophisticated and compact, and boast numerous superior features when compared to their predecessors. Within this sphere of practical research serving humankind, nanotechnology is no exception. It spans from thin films to nanowires and quantum dots, all of which engender remarkable applications in today’s technological realm.

Graphene, an exemplar material, possesses a hexagonal honeycomb structure and has undergone extensive theoretical and experimental investigations. This two-dimensional material constitutes a flat structure with six carbon (C) atoms situated at the hexagon vertices. Owing to the exceptional attributes of graphene, such as superior electrical and thermal conductivity, remarkable durability and ultra-lightweight characteristics, it has found extensive application across domains including energy storage, sensors, transistors, biomedicine and various other sectors [1–5]. Presenting strong contenders to graphene are germanene (Ge) [6–9] and silicene (Si) [10–13], promising to unlock myriad potential applications in the future. A shared characteristic among them is that the elements C, Si and Ge belong to group 4 within the periodic table of chemical elements and all share the hexagonal honeycomb structure. The distinction lies in the structural buckling of Si and Ge, arising from sp^2^ and sp^3^ hybridization [14,15]. It is believed that Ge possesses a buckling structure with a buckling height of approximately 0.61 Å. Owing to this structural feature, Ge readily engages in chemical reactions with hydrogen, leading to a structure with a wider bandgap, thereby impacting its electronic properties [6]. Density Functional Theory (DFT) research reveals that Ge is a semimetal with no energy gap, although surface functionalization with H, F or Cl [16,17] can extend its energy gap. The research also demonstrates that H functionalization yields a wider bandgap than F or Cl functionalization. Moreover, H functionalization results in ferromagnetic semiconductor systems, while F and Cl functionalization yields antiferromagnetic semiconductor systems [16]. The introduction of dopants or adsorbents into Ge can lead to novel materials with diverse superior characteristics, diversifying their potential applications [18–22].

Minglei Sun *et al*. have explored the doping of transition metals into two-dimensional Ge monolayers [23], revealing the emergence of magnetism upon doping with V, Cr, Mn, Fe and Co. Notably, even minute quantities of Mn doping in Ge yield substantial magnetic moment (approximately 3.5 µ_B_), suggesting the possibility of using Mn as a dopant to manipulate the magnetism of the system. The study of Al doping into Ge, conducted through a first-principles approach, has shown that the Al doping process enhances light reflection and absorption from Ge [20]. When fully hydrogenated, the Al doping configuration in Ge alters the energy band structure, shifting from an indirect bandgap to a direct bandgap. Additionally, DFT research has revealed that CO_2_, H_2_S and SO_2_ gas adsorption processes are suitable for potential gas sensors [19], with different levels of sensitivity. Defects in the Ge structure enhance the adsorption sensitivity to these gases. Recent years have seen increasing research attention towards another low-dimensional structure of Ge, known as one-dimensional Ge structures or Ge nanoribbons [24–28]. Functionalizing the edges of Ge nanoribbons brings about significant changes in the bandgap of the system. Armchair Ge nanoribbons exhibit energy gap widths of 0.012 and 0.84 eV when edges are functionalized by -2H and -2F, respectively. In cases of N or B doping in zig-zag Ge nanoribbons, the system transitions from antiferromagnetic semiconducting to ferromagnetic or semimetallic semiconducting [29]. The research on C and Si doping in armchair Ge nanoribbons has illuminated potential applications in optoelectronic devices and transistors [30]. The hydrogen-functionalized pristine configuration demonstrates semiconducting properties, characterized by a buckling height of 0.73 Å. The doping configuration with 50% C results in a structure with an expanded energy gap of up to 1.91 eV. This study leverages DFT to scrutinize the one-dimensional structure of CGe. The adsorption of N, helium (He) and neon (Ne) gases onto CGe nanoribbons aims to reveal the structural and electro-optical properties of these adsorption configurations. Despite having fully filled electron configurations, both He and Ne are considered light elements. This characteristic suggests that they can be readily adsorbed onto the surface of CGe nanoribbons for gas sensing applications. Furthermore, owing to their lightweight nature, He and Ne are anticipated to exert a minimal influence on the hexagonal structure of CGe nanoribbons.

## 2. Research methods

The theoretical underpinnings of this research are rooted in the DFT and simulations are executed using the Vienna ab initio Simulation Package (VASP) [31]. Within the framework of the general gradient approximation (GGA), the Perdew–Burke–Ernzerhof (PBE) pseudopotential [32] is used. Key input parameters encompass a cutoff energy of 500 eV, with EDIFF = 10^−7^ eV signifying the energy convergence threshold between successive ground-state steps and EDIFFG = 0.01 eV/Å serving as the Hellman–Feynman force limit. During the structural optimization phase, a KPOINTS grid of 1 × 1 × 11 is initially used. Subsequently, for structural property calculations, the KPOINTS grid is extended to 1 × 1 × 100. To correct interactions between monolayers, a vacuum spacing of 20 Å is introduced. The adsorption energy of the configurations is computed using the following equation [33]:


(2.1) $$E_{A}=E_{t}-E_{p}-E_{X},$$

where E_t_ and E_p_ denote the total energy of the adsorbed configuration and the pristine configuration, respectively, while E_X_ represents the energy of the adsorbed gas (N, He or Ne). The dielectric function of the system comprises two constituents: the real part and the imaginary part:


(2.2) $$\varepsilon \left(\omega \right)=\varepsilon_{1}\left(\omega \right)+i\varepsilon_{2}\left(\omega \right).$$

Here, $\varepsilon_{1}(\omega )$ signifies the real component, while $\varepsilon_{2}(\omega )$ represents the imaginary component. Various other optical properties, such as the absorption coefficient and refractive index, can be derived from the dielectric function.

The investigated CGe nanoribbon structures possess a width of six atoms, comprising three C atoms and three Ge atoms arranged in an evenly spaced manner, ensuring the absence of adjacent C (or Ge) atoms. Consequently, each unit cell comprises 12 atoms, with 6 C atoms, 6 Ge atoms, along with 4 H atoms functionalized at the two edges.

## 3. Results and discussion

### 3.1. Structural characteristics and electrical properties

**Figure 1.**
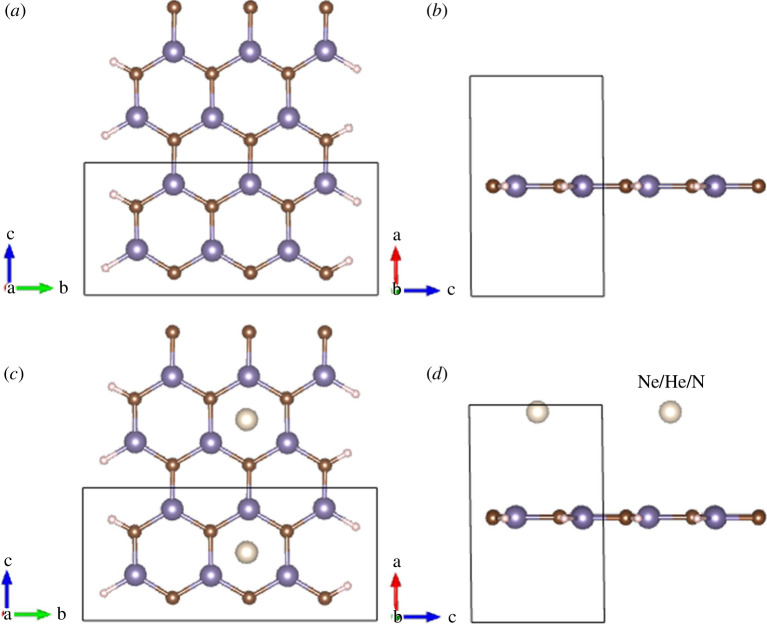
Top view (*a*) and side view (*b*) of the pristine configuration. Top view (*c*) and side view (*d*) of the adsorption configurations (C and Ge atoms are copper and gray, respectively. The white atom is the gas Ne, He or N and the hydrogen atoms are functionalized on both sides).

Figure 1 illustrates both the top view and side view of the configurations under investigation. The pristine configuration consists of a six-atom width (comprising three Ge atoms and three C atoms) with two edges functionalized by hydrogen atoms. Post-optimization, the configuration of CGe nanoribbons is a planar structure, highlighting the role of C in achieving structural flatness. The adsorption structures exhibit stability and maintain the honeycomb hexagonal pattern even after optimization.

**Figure 2.**
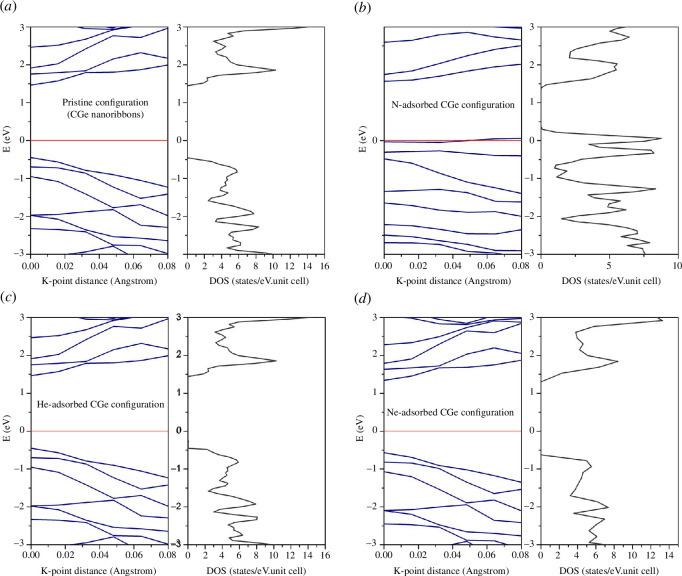
Band structures and density of states of the (*a*) pristine configuration, (*b*) N-adsorbed configuration, (*c*) He-adsorbed configuration and (*d*) Ne-adsorbed configuration.

Table 1 presents the state parameters for both the pristine configuration and adsorption configurations. It is evident that the pristine configuration is a semiconductor, featuring a bandgap of 1.93 eV. Upon adsorbing He and Ne, the semiconductor properties of the system remain largely unaltered, with only a minimal shift in the energy gap (as depicted in figure 2). Notably, N adsorption leads to the loss of semiconductor characteristics, transforming the system into a semimetal rich in properties (where the spin-up state exhibits semiconductor traits and the spin-down state showcases metallic attributes). The adsorption energy, which gauges the feasibility of the adsorption process, is detailed in table 1. Notably, both N and He adsorption energies yield positive values, while Ne adsorption energy is negative. Positive values indicate a relatively challenging adsorption process, implying an endothermic system. Consequently, the N adsorption process is the most challenging among the gases considered. Magnetic properties, at both the atomic and overall system levels, are expressed through the magnetic moment (μ), representing the total magnetic moment in a unit cell. Table 1 reveals that after adsorbing He and Ne, the system becomes non-magnetic, whereas N adsorption has the opposite effect. The N adsorption configuration significantly alters the system’s magnetism, resulting in a substantial magnetic moment of approximately 0.588 μ_B_. Table 1 also provides insight into the total charge within the Wigner–Seitz sphere. In comparison to the pristine system, gas adsorption leads to an increase in the charge within this sphere. Remarkably, Ne adsorption results in the most substantial charge increase, which aligns with the expectations, given Ne’s possession of eight outer shell electrons capable of facilitating a pronounced shift in charge density.

**Table 1.** Some structural parameters of the configurations (E_A_ is the adsorption energy, μ is the magnetic moment and the total charge is the total charge in the Wigner–Seitz sphere).

configurations	bandgap (eV)	E_A_ (eV)	μ (μ_B_)	total charge (e)	semiconductor/metal/semimetal
pristine	1.93	x	0	32.754	semiconductor
N adsorption	x	0.95	0.588	36.123	semimetal
He adsorption	1.91	0.01	0	34.190	semiconductor
Ne adsorption	1.92	−0.03	0	40.184	semiconductor

The Fermi level of the pristine configuration is situated at −4.4405 eV. When calculating the energy band structure and density of states, the reference point for energy levels is defined as E−E_F_ = 0. The semiconducting and semimetallic characteristics of the configurations are visually depicted within the energy band structure (figure 2). With substantial energy bandgaps measuring 1.93, 1.91 and 1.92 eV for the pristine, He-adsorbed and Ne-adsorbed configurations, respectively, these structures are exceptionally well-suited for optoelectronic and gas sensing applications. Upon examining figure 2, it becomes evident that the energy band structure and density of states for the pristine configuration and the two doped configurations, Ne and He, exhibit remarkable similarities. This observation underscores the limited impact of He and Ne on the electrical properties of the system, primarily due to their lightweight gaseous nature, which exerts a minimal influence on the CGe structure. In contrast, N doping leads to the loss of the semiconducting properties of the system, resulting in emergent states concentrated around the zero energy level, with numerous state peaks appearing in this region.

**Figure 3.**
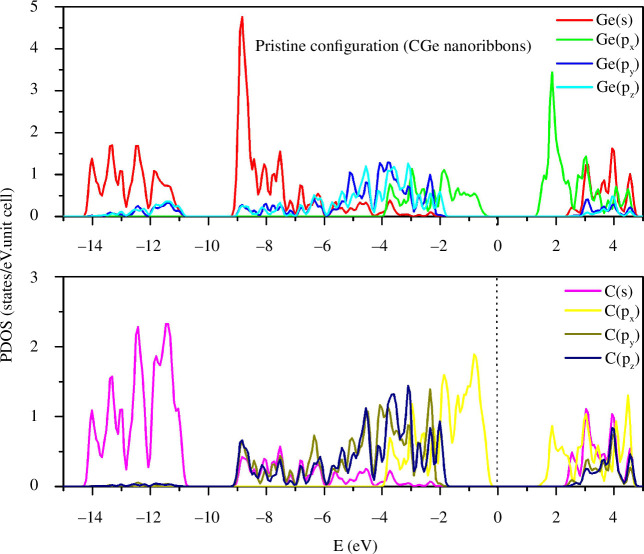
PDOS of the pristine configuration.

The partial density of states (PDOS) for the pristine configuration is depicted in figure 3. The s-state exhibits peaks concentrated in the deep energy region, considerably distant from zero. Meanwhile, the p(x), p(y) and p(z) states reveal their peaks clustered around the zero energy level, with the largest peaks predominantly belonging to the s-state. Specifically, the most prominent Ge(s) peak resides at an energy level of −8.8 eV, while the primary C(s) peak is situated at −11.2 eV. The intricate multi-orbital hybridizations are notably concentrated in the energy range spanning from −8 to −2 eV, as well as from 2 to 5 eV. The equilibrium in the density of both C and Ge particles contributes to a harmonious distribution of their respective states across both the conduction and valence bands.

**Figure 4.**
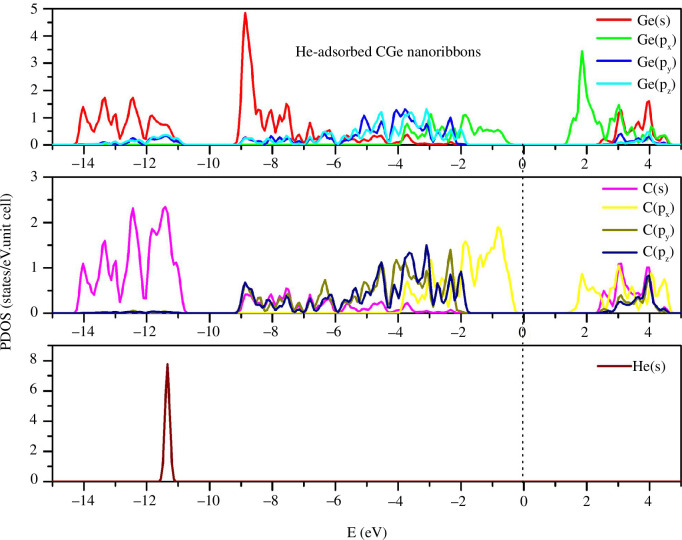
PDOS of the He-adsorbed configuration.

In the He adsorption configuration (figure 4), the PDOS of C and Ge remains largely unaltered in comparison with the pristine configuration. The impact of He on the electronic structure of the system is minimal. This can be attributed in part to the fact that He possesses only two electrons in the s subshell, resulting in its relatively inconsequential contribution. Notably, the s-states of He are predominantly concentrated in the deep energy region, within the energy range of −11 to 11.5 eV. There are no He states observed in the vicinity of the E = 0 level. Furthermore, given the absence of He states in the conduction band, He’s influence on shaping the electrical properties of the system is negligible.

**Figure 5.**
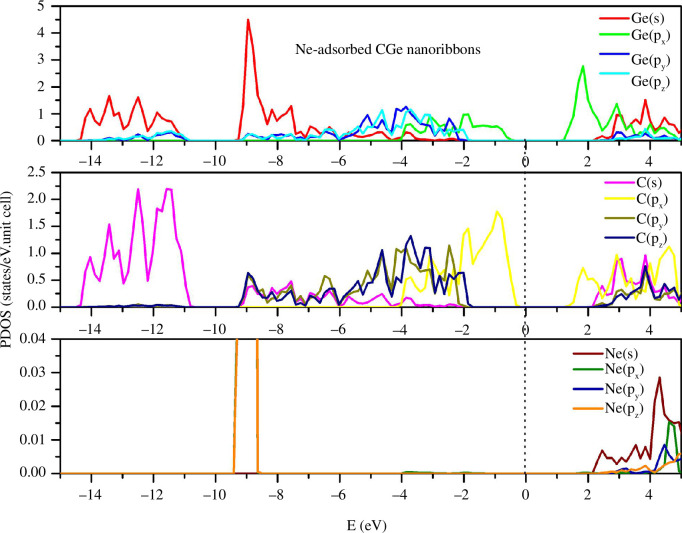
PDOS of the Ne-adsorbed configuration.

A more pronounced distinction emerges with the Ne doping configuration (figure 5). Although the distribution of states C(s, p_x_, p_y_, p_z_) and Ge(s, p_x_, p_y_, p_z_) from the top to the valence band remains relatively consistent, the state peaks exhibit alterations. Specifically, the highest peaks of Ge(s) and C(s) in the pristine configuration are 4.8 and 2.3, respectively. In the Ne-doped configuration, these peak values are altered to 4.5 and 2.2, respectively. Furthermore, the most prominent peak of Ge(p_x_) in the pristine configuration, with a value of 3.5, experiences a reduction to 2.7 in the Ne adsorption configuration. The multi-orbital hybridizations are predominantly concentrated within the conduction band, particularly in the energy range spanning from 2 to 5 eV.

**Figure 6.**
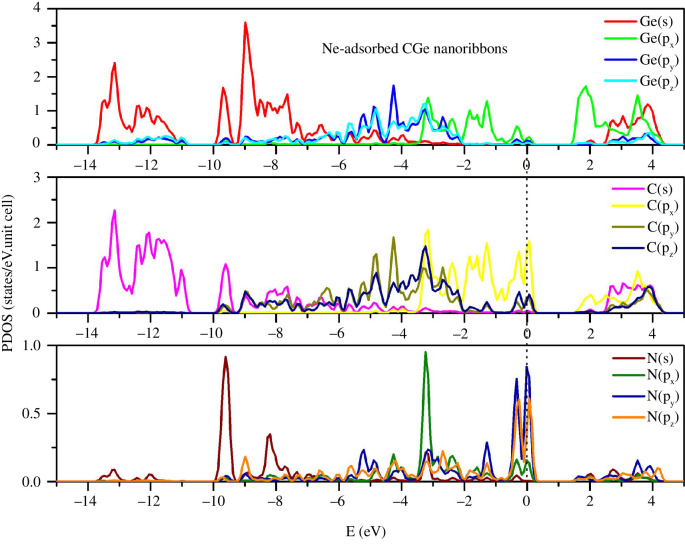
PDOS of the N-adsorbed configuration.

In the case of the N adsorption process (figure 6), a notable distinction is the absence of states at energy levels below −14 eV and above 4.5 eV. However, numerous states are observed around the zero energy level. The multi-orbital hybridization within the system is highly intricate, spanning from the conduction band to the valence band. As an example, s-p hybridization takes place within the energy range of 2.5 to 4.4 eV, involving Ge(s)–C(p_y_,p_z_) orbitals. Additionally, C(s)–Ge(p_x_, p_y_, p_z_)–N(p_x_, p_y_, p_z_) hybridization occurs in the range of −0.5 to 0.4 eV and between −10 and −9.2 eV and so on. Despite the significant impact of N compared to the gases Ne or He, its contribution to the states remains moderate due to the low density of N per unit cell. All state peaks are situated at levels below 1.

**Figure 7.**
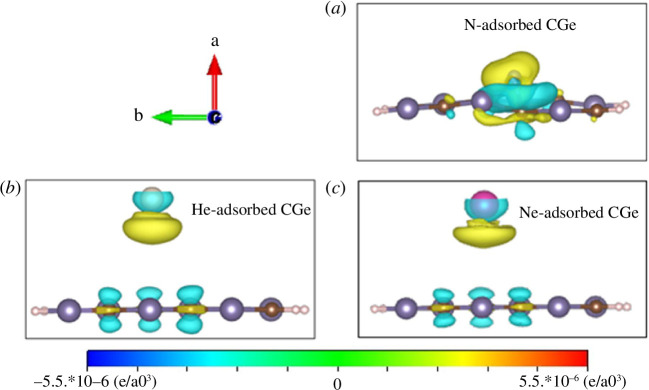
The charge density difference in configurations: the (*a*) N-adsorbed configuration, (*b*) He-adsorbed configuration and (*c*) Ne-adsorbed configuration (the blue region represents charge enhancement, while the yellow region represents charge depletion).

The variation in the charge density in these configurations is illustrated in figure 7. The two adsorption configurations for He and Ne share similarities, given that both elements are light and the adsorption site is relatively distant from the CGe surface. The distances from N, He and Ne to the CGe surface are 1.5, 4.86 and 4.92 Å, respectively. The considerable distance from the adsorbent surface results in charge redistribution primarily around the He and Ne atoms. Specifically, the region facing the adsorption plane experiences charge loss (indicated by the yellow area), while the opposite region exhibits charge concentration (depicted by the blue area). This redistribution of charge around the He and Ne atoms also induces a redistribution of charge on the CGe surface. Areas with opposite charges tend to attract each other and this charge distribution mainly affects the Ge and C atoms in proximity to He/Ne. In contrast, with N adsorption, N attaches very closely to the CGe surface. Consequently, the structure of the CGe surface appears slightly curved, with the curved part facing N. A charge shift occurs from N towards the CGe surface, and this shift is responsible for generating the complex multi-orbital hybridizations discussed earlier.

### 3.2. Optical properties

**Figure 8.**
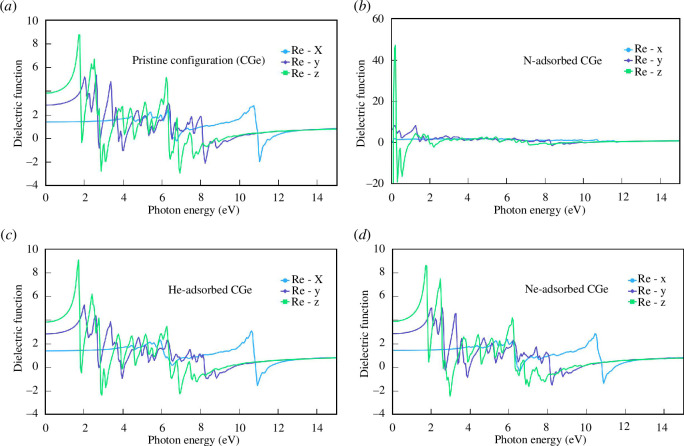
The real part of the dielectric function of the configurations: (*a*) pristine configuration, (*b*) N-adsorbed configuration, (*c*) He-adsorbed configuration and (*d*) Ne-adsorbed configuration.

The analysis of the real part of the dielectric function provides insights into the phase delay of the incident and reflected wave frequencies, shedding light on the energy storage capacity of the material under investigation (figure 8). Notably, all four configurations under study exhibit a range of negative values in the real part of the dielectric function. A negative value in the real part of the dielectric function signifies the unique properties of these materials, indicating that they allow electromagnetic waves to readily propagate through them. These findings affirm the suitability of these materials for applications in information and communication, offering minimal signal loss.

The real part of the dielectric function in the He/Ne adsorption configurations closely resembles that of the pristine configuration, suggesting that He/Ne has a limited influence on the transmission of electromagnetic waves in doped systems. However, a significant distinction arises with the N adsorption configuration. In the energy region below 1 eV, the dielectric constant assumes notably high values, particularly in the 0z direction, which represents the direction of free electron movement. As the energy exceeds 1.8 eV, the dielectric constant decreases, facilitating easier transmission of radiation through the material. This energy range corresponds to the region of visible light and beyond.

**Figure 9.**
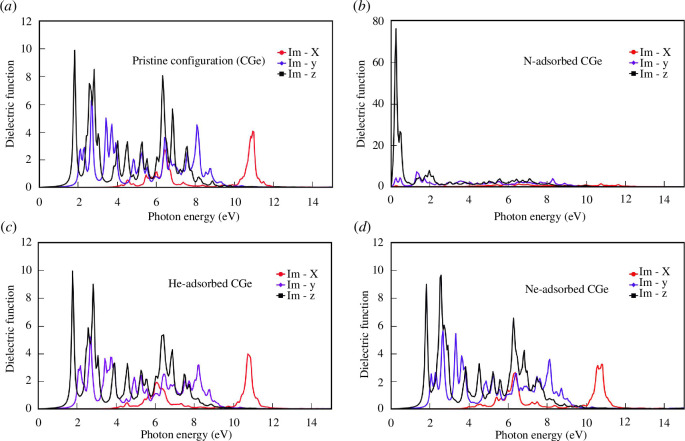
The imaginary part of the dielectric function of the configurations: (*a*) pristine configuration, (*b*) N-adsorbed configuration, (*c*) He-adsorbed configuration and (*d*) Ne-adsorbed configuration.

The imaginary part of the dielectric function characterizes the absorption or dissipation of electromagnetic wave energy as it traverses the material. Similar to the real part of the dielectric function, the imaginary part graph of the dielectric function for the pristine configuration and the He/Ne adsorption configurations exhibits a nearly identical shape (figure 9). This observation reaffirms the minimal contribution of He and Ne to the optical properties of the material.

In both the pristine and He adsorption configurations, the largest peak is situated at an energy level of 1.9 eV in the 0z direction. However, in the case of the Ne adsorption configuration, this peak shifts to 2.7 eV. The most significant disparity arises with the N adsorption configuration, where the largest peak occurs at a remarkably low energy level of about 0.4 eV.

Optical anisotropy manifests within the energy range of 1 to 12 eV for the pristine and He/Ne-doped configurations, while in the case of N adsorption, it extends from 0 to 12 eV. Beyond the energy levels exceeding 12 eV, the electromagnetic wave experiences minimal damping in all three directions, indicating that the electromagnetic energy is sufficiently high to move freely within the material.

Notably, across all four configurations, the largest peaks are consistently found in the 0z direction, which aligns with the absence of size constraints in this direction. These peaks correspond to energy level transitions between states, specifically the transitions from C(2s) to Ge(3d, 4p) or from C(2p) to Ge(3d).

**Figure 10.**
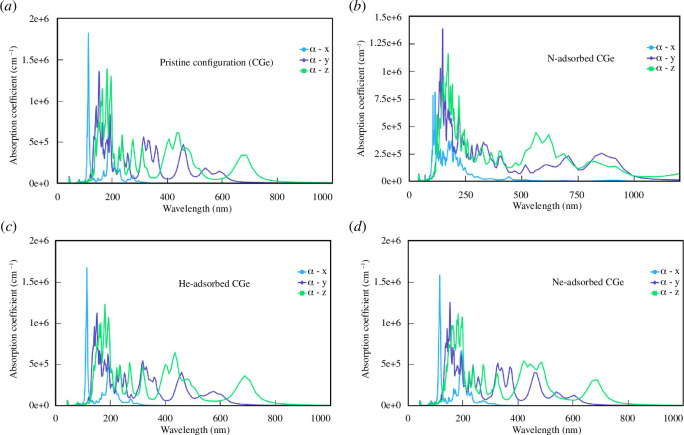
Absorption coefficient of the configurations:(*a*) pristine configuration, (*b*) N-adsorbed configuration, (*c*) He-adsorbed configuration and (*d*) Ne-adsorbed configuration.

The absorption coefficient quantifies the amount of light absorbed by the material per unit thickness. Figure 10 illustrates the absorption coefficient for various configurations. An examination of the graph reveals that the absorption coefficient also serves as an indicator of optical anisotropy for all three structures. The absorption peaks are predominantly situated within the short wavelength range, gradually diminishing at longer wavelengths. Specifically, for the pristine configuration and the He/Ne adsorption configurations, the absorption coefficient approaches zero when the wavelength exceeds 800 nm. Consequently, in these configurations, light achieves optical transparency when its wavelength exceeds 800 nm.

However, the N adsorption configuration exhibits different behaviour. In most wavelength ranges, the absorption coefficient remains at or near zero. Notably, the absorption coefficient in the 0x direction is consistently lower than those in the other directions for each wavelength. This observation suggests that the 0x direction exhibits the least absorption of electromagnetic waves. Given that the 0x direction consists of just one atom’s thickness, it absorbs electromagnetic waves to a lesser extent. Additionally, the highest peaks of the absorption coefficient are associated with the 0z direction in the pristine and He/Ne adsorption configurations, while in the N adsorption configuration, the highest peak corresponds to the 0y direction.

**Figure 11.**
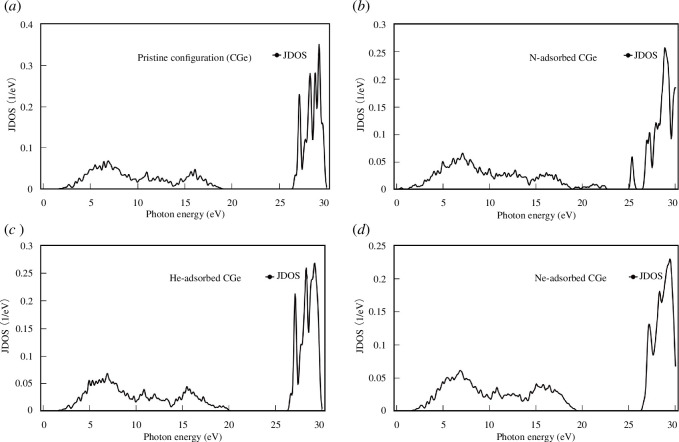
The JDOS of the configurations:(*a*) pristine configuration, (*b*) N-adsorbed configuration, (*c*) He-adsorbed configuration and (*d*) Ne-adsorbed configuration.

The Joint Density of States (JDOS) serves as a representation of the number of electron–hole pairs at specific energy levels, as illustrated in figure 11 for the studied configurations. Notably, it becomes evident that energies below 1.94 eV in both the pristine configuration and the He/Ne adsorption configuration do not yield electron–hole pairs. This aligns with the theoretical expectations, as electrons transition to the conduction band only when their energy surpasses the energy gap.

In contrast, the N adsorption configuration exhibits a sharp contrast. Being a semimetal, it imposes no restrictions on photon energy for electron–hole pair generation. However, it is important to note that not all high-energy photons produce electron–hole pairs. For instance, in the He adsorption configuration, photons with energies ranging from 20 to 26.3 eV, as well as those exceeding 30 eV, fail to create any electron–hole pairs. Across all configurations, the most prominent JDOS peaks are situated within the energy range of 27.5 to 30 eV.

Upon scrutinizing the aforementioned analysis, it is evident that in the examined configurations, the He/Ne adsorption configuration exhibits minimal deviation from the pristine configuration, indicating a negligible impact of He/Ne on the doping system. In contrast, the N adsorption configuration induces alterations in electrical, magnetic and optical properties. Notably, N instigates fundamental changes in the inherent properties of the initial system.

## 4. Conclusions

This research conducts a comprehensive investigation into the structural, electromagnetic and optical characteristics of CGe nanoribbons and CGe structures when adsorbed with N, He and Ne. The key findings offer an in-depth exploration of the electrical, magnetic and optical attributes of both the pristine and adsorbed systems. The pristine system presents itself as a semiconductor, featuring a bandgap of 1.93 eV. Likewise, the He and Ne adsorption systems exhibit semiconducting traits with bandgaps of 1.91 and 1.92 eV, respectively. Conversely, the N adsorption system exhibits semimetallic properties. Within this system, the spin-up state demonstrates semiconductor characteristics, while the spin-down state manifests metallic properties. The study further systematically investigates multi-orbital hybridization states, charge density variations and fundamental quantities in the optical properties of the configurations. The research indicates that the N adsorption configuration is fundamentally distinct from the others, causing a profound transformation in the electrical, magnetic and optical properties of the pristine system. Based on these remarkable characteristics, this research holds promise for applications in optoelectronic devices, sensors and future information transmission technologies.

## Data Availability

Data available from the Dryad Digital Repository [34].
